# Determinants of methicillin-susceptible *Staphylococcus aureus*native bone and joint infection treatment failure: a retrospective cohort study

**DOI:** 10.1186/1471-2334-14-443

**Published:** 2014-08-16

**Authors:** Florent Valour, Anissa Bouaziz, Judith Karsenty, Florence Ader, Sébastien Lustig, Frédéric Laurent, Christian Chidiac, Tristan Ferry

**Affiliations:** Service des maladies infectieuses et tropicales, Hospices Civils de Lyon, Groupement Hospitalier Nord, Lyon, France; Université Claude Bernard Lyon 1, INSERM U1111, International Centre for Research in Infectious diseases, Lyon, France; Chirurgie orthopédique, Hospices Civils de Lyon, Groupement Hospitalier Nord, Lyon, France; Laboratoire de bactériologie, Centre National de Référence des Staphylocoques, Hospices Civils de Lyon, Lyon, France; the Lyon BJI study group, France

**Keywords:** *Staphylococcus aureus*, Bone and joint infection, Treatment failure

## Abstract

**Background:**

Although methicillin-susceptible *Staphylococcus aureus* (MSSA) native bone and joint infection (BJI) constitutes the more frequent clinical entity of BJI, prognostic studies mostly focused on methicillin-resistant *S. aureus* prosthetic joint infection. We aimed to assess the determinants of native MSSA BJI outcomes.

**Methods:**

Retrospective cohort study (2001–2011) of patients admitted in a reference hospital centre for native MSSA BJI. Treatment failure determinants were assessed using Kaplan-Meier curves and binary logistic regression.

**Results:**

Sixty-six patients (42 males [63.6%]; median age 61.2 years; interquartile range [IQR] 45.9–71.9) presented an acute (n = 38; 57.6%) or chronic (n = 28; 42.4%) native MSSA arthritis (n = 15; 22.7%), osteomyelitis (n = 19; 28.8%) or spondylodiscitis (n = 32; 48.5%), considered as “difficult-to-treat” in 61 cases (92.4%). All received a prolonged (27.1 weeks; IQR, 16.9–36.1) combined antimicrobial therapy, after surgical management in 37 cases (56.1%). Sixteen treatment failures (24.2%) were observed during a median follow-up period of 63.3 weeks (IQR, 44.7–103.1), including 13 persisting infections, 1 relapse after treatment disruption, and 2 super-infections. Independent determinants of treatment failure were the existence of a sinus tract (odds ratio [OR], 5.300; 95% confidence interval [CI], 1.166–24.103) and a prolonged delay to infectious disease specialist referral (OR, 1.134; 95% CI 1.013–1.271).

**Conclusions:**

The important treatment failure rate pinpointed the difficulty of cure encountered in complicated native MSSA BJI. An early infectious disease specialist referral is essential, especially in debilitated patients or in presence of sinus tract.

**Electronic supplementary material:**

The online version of this article (doi:10.1186/1471-2334-14-443) contains supplementary material, which is available to authorized users.

## Background

Bone and joint infections (BJIs) constitute difficult-to-treat clinical entities, known to be associated to significant morbidity and mortality rates. Most of the current literature on BJI concerns orthopaedic device infections and/or methicillin-resistant *Staphylococcus aureus* (MRSA). However, native infections represent the most frequent clinical form of BJI, accounting for approximately 70% of cases, and are mainly caused by methicillin-susceptible *Staphylococcus aureus* (MSSA) [1]. With a respective incidence of 4–10, 10 and 2.4 per 100,000 person-year, septic arthritis, osteomyelitis and vertebral osteomyelitis are associated with a mortality rate of 2-10%, and a risk of permanent loss of joint function of 40% [2, 3]. It has recently been shown that the setting of a systematic infectious disease specialist consultation in a septic orthopaedic surgery unit allows a better adjustment of empirical antimicrobial therapy [4]. However, risk factors for treatment failure have poorly been studied. We addressed this question in a retrospective cohort study.

## Methods

All patients with native MSSA BJI were enrolled in a monocentric retrospective cohort study (2001–2011) in the reference center for the management of complex BJI of the Lyon University Hospitals, France. To be included, patients should present clinical evidences of infection and at least one reliable bacteriological sample positive for MSSA including percutaneous joint fluid aspiration, surgical sample, and/or blood culture, excluding patients with diabetic foot- and decubitus ulcer-related BJI because of the specific management of these infections. The time from initiation of symptoms of infection to diagnosis defined acute (infection lasting for ≤ 4 weeks) and chronic (infection lasting for > 4 weeks) infections [5]. The modified Charlson comorbidity index was calculated as previously described [6]. Immunosuppression was defined as: i) steroid therapy > 10 mg of prednisone per day or equivalent; ii) immunosuppressive drug during the two last months before BJI onset; or iii) chemotherapy. Treatment failure included i) persisting infection under appropriate antimicrobial therapy; and/or ii) relapse after antimicrobial therapy disruption.

Data were collected from medical records, nursing charts and biological software in an anonymous standardized case report form. Frequencies of the study variables were described as effectives (%) for dichotomous variables, and medians (interquartile range [IQR]) for continuous values. For the percentage calculation of each variable, the number of missing values was excluded from the denominator. Non-parametric statistical methods were used to compare the study groups (Khi2, Fisher exact test, Mann–Whitney U test), as appropriate. Kaplan-Meier curves were compared between groups using the log-rank test. Stepwise binary logistic regression was used to determine risk factors for treatment failure. After checking variables for interactions, variables with medical meaning and p values obtained in univariate analysis < 0.15 were included in the final multivariate model. A value of p < 0.05 was taken as significant. All analyses were performed using SPSS software version 17.0 (SPSS, Chicago, IL).

This study received the approval of the French South-East ethics committee with the reference number CAL2011-21. In accordance with the French legislation, written informed patient consent was not required for any part of the study.

## Results

After exclusion of 4 diabetic foot- or decubitus ulcer-related infections and 7 patients with numerous missing values, 66 patients were enrolled in the analysis (42 males; 63.6%), with a median age of 61.2 years (IQR, 45.9–71.9). Demographic characteristics, comorbidities and BJI presentation are summarized in Table 1. Of note, 61 (92.4%) of included BJI were considered as difficult-to-treat, including chronic BJI (n = 28; 42.4%), local abscess (n = 33; 50.0%), sinus tract (n = 18; 27.3%), bacteraemia (n = 35; 53.0%) and/or associated infective endocarditis (n = 4; 6.1%). Importantly, in comparison with arthritis, osteomyelitis were more often chronic (n = 16 (84.2%) versus n = 2 (13.3%); p < 10^−3^), and sinus tract (n = 13 (68.4%) versus n = 2 (13.3%); p = 0.002) and abscesses (n = 10 (52.6%) versus n = 2 (13.3%); p = 0.030) were more frequent.

**Table 1:** **Patient’s characteristics and risk factors for native methicillin-susceptible** ***Staphylococcus aureus*** **bone and joint infection treatment failure**

Risk factor for treatment failure			Total (n = 66)	Treatment failure (n = 16)	Favourable outcome (n = 50)	p	Univariate analysis	
							OR (95%CI)	p
**Demographic characteristics**								
	Sex (male)		42 (63.6%)	10 (62.5%)	32 (64.0%)	0.913	0.938 (0.292-3.006)	0.938
	Age (years)		61.2 (45.9-71.9)	61.2 (48.5-69.6)	60.4 (43.5-76.5)	0.828	1.119 (0.820-1.525)*	0.479
**Comorbidity**								
	Modified Charlson score		3.0 (0.0-5.0)	4.0 (2.5-5.0)	2.0 (0.0-4.0)	0.163	1.093 (0.907-1.318)	0.351
	Modified Charlson score > 2		34 (51.5%)	12 (75.0%)	22 (44.0%)	0.044	3.818 (1.081-13.486)	0.037
	Obesity (BMI > 30 kg/m^2^)		13 (20.0%)	3 (20.0%)	10 (20.0%)	1.000	1.000 (0.236-4.231)	1.000
	Denutrition (BMI < 18 kg/m^2^)		3 (4.6%)	1 (6.7%)	2 (4.0%)	1.000	1.714 (0.145-20.332)	0.669
	Diabetes		11 (16.7%)	6 (37.5%)	5 (10.0%)	0.018	5.400 (1.372-21.260)	0.016
	Immunodepression		8 (12.1%)	3 (18.8%)	5 (10.0%)	0.390	2.077 (0.437-9.871)	0.358
	Nephropathy		10 (15.2%)	3 (18.8%)	7 (14.0%)	0.695	1.418 (0.320-6.277)	0.646
	Hepatopathy		2 (3.0%)	2 (12.5%)	0 (0%)	0.056	NC	NC
	Chronic pulmonary disease		12 (18.2%)	4 (25.0%)	8 (16.0%)	0.465	1.750 (0.449-6.825)	0.420
	Chronic heart failure		5 (7.6%)	0 (0%)	5 (10.0%)	0.325	NC	NC
	Chronic inflammatory disease		7 (10.6%)	2 (12.5%)	5 (10.0%)	1.000	1.286 (0.224-7.370)	0.778
	Neoplasm, hemopathy		7 (10.6%)	1 (6.3%)	6 (12.0%)	0.674	0.489 (0.054-4.397)	0.523
	Dementia		1 (1.5%)	1 (6.3%)	0 (0%)	0.242	NC	NC
**BJI type**								
	Arthritis		15 (22.7%)	3 (18.8%)	12 (24.0%)	0.747	0.731 (0.178-3.003)	0.731
	Osteomyelitis		19 (28.8%)	8 (50.0%)	11 (22.0%)	0.054	3.545 (1.082-11.615)	0.037
	Vertebral osteomyelitis		32 (48.5%)	5 (31.3%)	27 (54.0%)	0.195	0.387 (0.117-1.279)	0.120
**BJI mechanism**								
	Haematogenous		40 (60.6%)	8 (50.0%)	32 (64.0%)	0.480	0.563 (0.180-1.754)	0.321
	Inoculation		22 (33.3%)	7 (43.8%)	15 (30.0%)	0.475	1.815 (0.570-5.779)	0.313
	Contiguity		4 (6.1%)	1 (6.3%)	3 (6.0%)	1.000	1.044 (0.101-10.806)	0.971
**BJI diagnosis**								
	Fever		43 (65.2%)	10 (62.5%)	33 (66.0%)	1.000	0.859 (0.267-2.764)	0.798
	Fistula		18 (27.3%)	7 (43.8%)	11 (22.0%)	0.112	2.758 (0.836-9.092)	0.096
	Abscess		33 (50.0%)	7 (43.8%)	26 (52.0%)	0.775	0.718 (0.231-2.229)	0.566
	Chronic BJI (evolution > 4 weeks)		28 (42.4%)	8 (50.0%)	20 (40.0%)	0.680	1.500 (0.484-4.651)	0.483
	Delay from symptoms to diagnosis (weeks)		2.1 (0.0-10.3)	2.6 (0.0-34.4)	2.1 (0.5-9.8)	0.905	1.026 (0.996-1.057)	0.095
	Polymicrobial BJI		10 (15.2%)	3 (18.8%)	7 (14.0%)	0.695	1.418 (0.320-6.277)	0.646
	Infective endocarditis		4 (6.1%)	0 (0%)	4 (8.0%)	0.565	NC	NC
	Biological inflammatory syndrome		58 (87.9%)	16 (100%)	42 (84.0%)	0.183	NC	NC
		Maximal CRP value (mg/L)	152.7 (52.0-317.8)	145.0 (75.3-317.3)	154.7 (52.0-325.9)	0.994	1.000 (0.996-1.004)	0.931
		Maximal WBC count value (/mm^3^)	10,200 (7,720-14,920)	11,000 (10,200-16,280)	9,710 (7,350-14,770)	0.100	1.058 (0.961-1.164)	0.251
		Maximal neutrophil count value (/mm^3^)	7,600 (5,200-11,970)	9,300 (6,740-13,470)	7,380 (5,200-11,400)	0.292	1.056 (0.957-1.165)	0.277
	Chronic sepsis on pathological examination		9/21 (47.4%)	2/3 (66.7%)	7/16 (43.8%)	0.582	2.571 (0.192-34.473)	0.476
**Surgical treatment**			37 (56.1%)	11 (68.8%)	26 (52.0%)	0.377	2.031 (0.615-6.701)	0.245
	Delay from symptoms to surgery (days)		3 (0–12.5)	0 (0–9)	4 (0–12)	0.402	0.999 (0.994-1.004)	0.632
**Antibiotic use**								
	Delay from diagnosis to specialist referral (days)		4.9 (0.0-23.1)	6.3 (0.7-91.0)	4.9 (0.0-18.5)	0.445	1.102 (1.003-1.211)	0.043
	i.v.treatment		59 (89.4%)	13 (81.3%)	46 (92.0%)	0.347	0.377 (0.075-1.901)	0.237
		i.v.treatment duration (weeks)	7.1 (4.9-11.7)	9.1 (5.3-16.4)	7.0 (4.6-9.4)	0.297	1.037 (0.986-1.091)	0.156
	Bitherapy		66 (100%)	16 (100%)	50 (100%)	1.000	NC	NC
		Bitherapy duration (weeks)	25.6 (15.0-32.1)	27.0 (17.4-38.5)	25.1 (15.1-31.1)	0.533	1.016 (0.983-1.050)	0.345
		Initial anti-staphylococcal bitherapy	53 (81.5%)	12 (75.0%)	41 (83.7%)	0.719	0.585 (0.150-2.285)	0.441
		Initial anti-MSSA bitherapy	40 (61.5%)	8 (50.0%)	32 (65.3%)	0.480	0.531 (0.169-1.666)	0.278
**Biological follow-up**								
	1 month CRP level		13.0 (3.6-36.0)	13.3 (7.4-70.4)	11.0 (3.2-31.9)	0.296	1.006 (0.994-1.018)	0.315
	Decrease in CRP level at 1 month < 50%		9 (13.8%)	4 (26.7%)	5 (10.0%)	0.204	3.273 (0.752-14.245)	0.114
	1 month CRP level < 10 mg/L		29 (44.6%)	5 (33.3%)	24 (48.0%)	0.377	0.542 (0.162-1.814)	0.320

Results are presented as n (%) for dichotomic variables compared using Chi-square or Fisher exact tests, and median (interquartile range) for continuous variables, compared using Mann–Whitney U-test. Risk factors for treatment failure were assessed using logistic binary regression.

*For a 10-year increase in age.

BJI, Bone and joint infection; CI, Confidence interval; BMI, Body mass index; CRP, C-reactive protein; i.v., Intravenous; MSSA, Methicillin-susceptible Staphylococcus aureus; NC, Not calculable; OR, Odds ratio; WBC, White blood cells.

A surgical management was performed in 37 cases (56.1%). All patients received antimicrobial therapy for 27.1 weeks (IQR, 16.9–36.1), initially administrated intravenously in 59 patients (89.4%) for 7.1 weeks (IQR, 4.9–11.7). All patients received a combined antistaphylococcal therapy during almost all treatment duration (25.6 weeks; IQR, 15.0-32.1). The antimicrobial were chosen according to recommendations and microbiological susceptibility testing in all cases, with respect of contraindications (i.e., drug interactions, previous adverse events …). The main used molecules, doses and duration are presented in Table 2. Of note, 25 patients (37.9%) received glycopeptides, given as initial empirical therapy (n = 4; for a total duration ≤ 14 days), a previous allergic reaction to other antistaphylococcal antibiotics (n = 12), a polymicrobial infection (n = 5), or difficult venous access (n = 4, then using subcutaneous teicoplanin).

**Table 2:** **Main antimicrobial used in the 66 included patients with native methicillin-susceptible** ***Staphylococcus aureus*** **native bone and joint infection**

		All patients (n = 66)	Treatment failure (n = 16)	Favorable outcome (n = 50)	p
i.v anti-staphylococcal penicillin		49 (74.2%)	11 (68.8%)	38 (76.0%)	0.743
	Dose (mg/kg/day)	144.6 (133.3-169.0)	141.2 (133.3-150.0)	144.9 (133.6-172.7)	0.606
	Duration (weeks)	6.0 (3.0-8.0)	6.7 (4.1-12.2)	5.2 (2.7-7.4)	0.250
Glycopeptides		25 (37.9%)	7 (43.8%)	18 (36.0%)	0.768
	Vancomycine,	10 (15.2%)	1 (6.3%)	9 (18.0%)	0.430
	Dose (mg/kg/day)	26.0 (20.3-30.5)	25	27.0 (19.2-31.3)	NC
	Teicoplanin	22 (33.3%)	6 (37.5%)	16 (32.0%)	0.764
	Dose (mg/kg/day)	5.7 (4.1-7.0)	7.2 (5.4-8.4)	5.2 (3.9-6.5)	0.197
	Duration	3.4 (2.6-7.6)	3.4 (1.9-16.1)	3.9 (2.8-6.7)	0.832
Aminoglycosides		38 (57.6%)	9 (56.3%)	29 (58.0%)	1.000
Rifampin		36 (54.5%)	9 (56.3%)	27 (54.0%)	1.000
	Dose (mg/kg/day)	18.8 (14.6-21.2)	18.0 (14.6-21.4)	18.8 (15.2-20.8)	0.841
	Duration (weeks)	20.3 (2.7-34.6)	27.5 (11.8-53.1)	16.0 (2.7-25.3)	0.334
Fluoroquinolones		62 (93.9%)	15 (93.8%)	47 (94.0%)	1.000
	Ofloxacin dose (mg/kg/day)	6.7 (5.8-7.5)	7.1 (6.3-7.5)	6.35 (5.7-7.3)	0.240
	Duration (weeks)	14.6 (8.0-27.6)	17.0 (10.9-31.9)	14.6 (7.6-24.4)	0.397
Macrolid group		46 (69.7%)	10 (62.5%)	36 (72.0%)	0.538
	Clindamycin	17 (25.8%)	6 (37.5%)	11 (22.0%)	0.324
	Pristinamycin	33 (50.0%)	5 (31.3%)	28 (56.0%)	0.150
Linezolid		6 (9.1%)	1 (6.3%)	5 (10.0%)	1.000
Fucidic acid		4 (6.1%)	1 (6.3%)	3 (6.0%)	1.000
Fosfomycin		13 (19.7%)	4 (25.0%)	9 (18.0%)	0.719
Cotrimoxazole		2 (3.0%)	1 (6.3%)	1 (2.0%)	0.429

i.v: intravenous.

Difference between the two groups were assessed using Chi-square test or Fisher exact test for dichotomic variables, and Mann–Whitney U-test for continuous variables.

Treatment failure was observed in 16 cases (24.2%) during a median follow-up period of 63.3 weeks (IQR, 44.7–103.1): i) 13 patients (19.7%) with persistent infection including 9 patients requiring new surgery performed in a delay of 11.6 weeks (IQR, 5.3–25.6) after antimicrobial treatment initiation; ii) one relapse occurring 13.7 weeks after treatment disruption; and iii) 2 super-infections (one with *Staphylococcus epidermidis*, and one with *Enterococcus faecalis* and *Streptococcus intermedius*). Final evolution was favourable in 12 of the 16 patients with initial treatment failure. Three patients had to be amputated. Five patients (7.6%) died during follow-up, without sepsis-related death. Of note, one fatal pulmonary embolism linked with prolonged bed rest occurred. At the end of follow-up, 24 patients (38.1%) presented functional sequels, consisting in chronic pain and/or loss of function.

**Figure 1:**
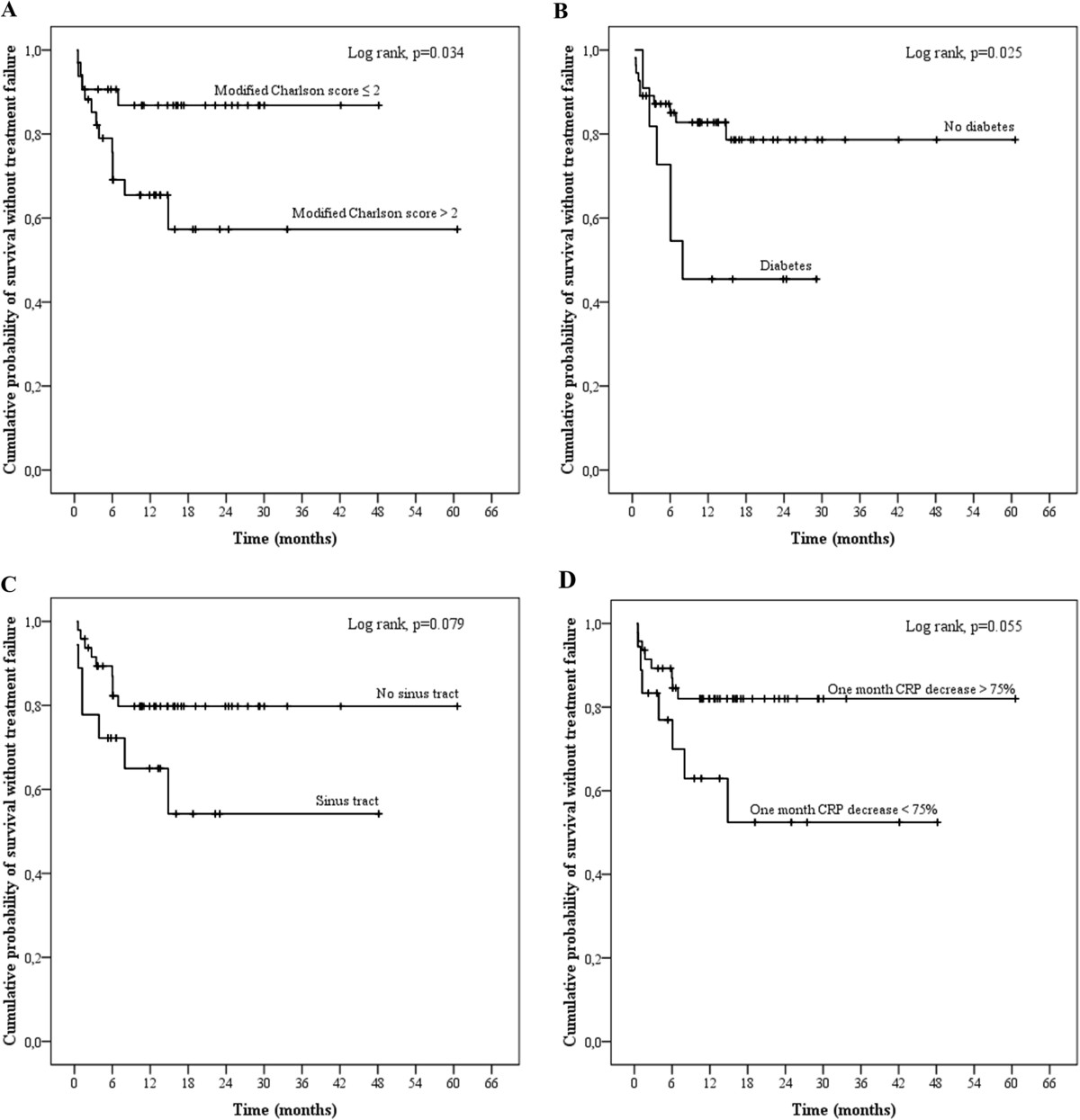
**Kaplan-Meier curves for the cumulative risk of treatment failure.** Kaplan-Meier curves for the cumulative risk of treatment failure are presented according to the modified Charlson comorbidity index (panel **A**), the presence of diabetes (panel **B**) or sinus tract (panel **C**), and the 1-month CRP level (panel **D**). Groups were compared using the log-rank test.

Patients presenting a treatment failure did not differ from those with favourable outcome regarding their baseline characteristics, with the exception of a higher prevalence of diabetes (37.5% versus 10%; p = 0.018) and a higher number of patients presenting a modified Charlson comorbidity index > 2 (75.0% versus 44.0%; p = 0.044). There was no difference between the two groups regarding the use of the main administered antimicrobials (Table 2). The delay from diagnosis to infectious disease specialist advice (i.e. first phone contact, consultation or hospitalization) tended to have been higher for patients with treatment failure (6.3 days; IQR, 0.7–91.0; p = 0.445), and especially for those with persistent infection (7.7 days; IQR, 0.4–161.0; p = 0.217) than for patients with favourable outcome. In univariate analysis, diabetes (Odd ratio [OR], 5.4; 95% confidence interval [CI], 1.372–21.260; p = 0.016), osteomyelitis (OR, 3.545; 95% CI, 1.082–11.615; p = 0.037), and a prolonged delay for infectious disease specialist referral (OR, 1.102; 95% CI, 1.003–1.211; p = 0.043) were associated with treatment failure (Table 1, Figure 1). Non-interacting and clinically relevant factors included in the multivariate logistic regression model were a modified Charlson comorbidity index > 2 (OR, 3.322; 95% CI, 0.753–14.661; p = 0.113), the existence of a sinus tract (OR, 5.300; 95% CI, 1.166–24.103; p = 0.031), a delayed referral to infectious disease specialist (OR, 1.134; 95% CI, 1.013–1.271; p = 0.029), and a decreased in CRP level at 1 month < 75% (OR, 3.183; 95% CI, 0.727–13.936; p = 0.124).

## Discussion

In this retrospective cohort study including patients with native MSSA BJI, we pinpointed an important rate of unfavourable outcome, including a treatment failure rate reaching one quarter of patients and high proportion of functional sequels. These results should be interpreted in light of the high prevalence of difficult-to-treat infections enrolled in the study, due to the particular recruitment of our institution, a reference centre for the management of complex BJI. Moreover, the implication of *S. aureus* is known to be associated with a poorer outcome of native and device-associated septic arthritis [7–9]. However, these findings are consistent with the few available data in the literature [2, 10]. Most of studies focusing on native staphylococcal BJI outcome were large epidemiologic investigations, based on national health surveillance programs, and consequently not design to assess precise outcome but only mortality. In a previous study, Wieland et al. disclosed a treatment failure rate of 12.2% among 41 native MSSA BJI [11]. Although these authors provided no detailed information about the type of patient recruitment, the short treatment court duration (43 days) and the low amount of patient requiring nursing home or rehabilitation facility (11%) allow supposing that common forms of BJI were more represented. In our particular patient population requiring long-term antimicrobial therapy, independent risk factors for treatment were the presence of a sinus tract and a delayed referral to infectious disease specialist. Fistula has already been associated with poor outcome in prosthetic-joint infection and vertebral osteomyelitis [12, 13]. Our findings confirmed that this clinical evidence for chronic infection is associated with treatment failure of native BJI. Diabetes was associated with a higher risk of treatment failure in univariate analysis but was excluded from the final model because the parameter was included in the Charlson comorbidity score calculation. However, it is a well-known risk factor for treatment failure [14]. Interestingly, we observed a trend in a higher treatment failure rate in bone infections (50.0%) compared to arthritis (18.8%). This difference probably lies in the higher rate of chronic infections, sinus tracts and abscesses among the osteomyelitis cases. Indeed, in the study by Wieland and colleagues, these two BJI types harboured the same outcome [11].

The impact of a referral to infectious disease specialist has been evaluated in several studies, which showed a benefit in terms of early adaptation of the initial empirical therapy after bacteriological results, and regarding dosages and duration of antimicrobials [4, 15]. However, these series failed to highlight an improvement of BJI outcome. Nevertheless, Bauer and colleagues showed a decrease from 25 to 18% of treatment failure rate after the instauration of a weekly multidisciplinary staff meeting in their institution, even if this difference was not significant [15].

Some studies had found other determinants of poor outcome in native BJI, including advanced age, a raised white cell count at presentation, the presence of an abscess, a delayed initiation of antimicrobial treatment, a pre-existing joint disease which may delay diagnosis [9, 13, 16–18]. We failed to found any association between outcome and the nature of antimicrobial therapy, and especially with the use of glycopeptides used as empirical therapy, for polymicrobial infection, or in patients with beta-lactam allergy. Indeed, vancomycin therapy has been associated with a poor outcome in MSSA bacteraemia, due to its slow bactericidal activity [19, 20]. One study including a majority of MSSA native osteomyelitis also suggested that vancomycin-treated infections were nearly three-times more likely to recur [21]. Contrary to prosthetic-joint infections, the use of rifampin did not appear as a protective factor in our study, possibly because of the less important implication of biofilm in absence of orthopaedic device. Finally, if the optimal treatment duration of BJI is unknown, a longer antimicrobial therapy did not appear as a protective factor. Prolonged antimicrobial therapy observed in our study is partly explained by the complicated nature of the included BJI. Another explanation lies in the retrospective nature of the study, which included patients in a 10-year period. Even in the absence of controlled randomized trial, the absence of evidence regarding the benefit of prolonged treatment lead to progressively decrease treatment duration in our population, without increasing failure rate over years (data not shown). Prospective controlled studies are needed to confirm the feasibility of shorter treatments. However, some studies had suggested that reducing treatment duration was associated with an increased risk of treatment failure, notably in vertebral osteomyelitis [22].

## Conclusions

MSSA native BJI are associated with a high rate of treatment failure and sequel, despite the use of prolonged antimicrobial therapy. A multidisciplinary approach is required, with an early referral to infectious disease specialist, especially in debilitated patients or in presence of a sinus tract.

## Authors’ original submitted files for images

Below are the links to the authors’ original submitted files for images. Authors’ original file for figure 1

## Abbreviations

BJI: Bone and joint infection
CI: Confidence interval
IQR: Interquartile range
MRSA: Methicillin-resistant *Staphylococcus aureus*
MSSA: Methicillin-susceptible *Staphylococcus aureus*
OR: Odds ratio.
