# Supplementary figures and images for: Determinants of methicillin-susceptible Staphylococcus aureusnative bone and joint infection treatment failure: a retrospective cohort study
Source: BMC Infect Dis. 2014 Aug 16;14:443. doi: 10.1186/1471-2334-14-443 (PMC4147168; doi:10.1186/1471-2334-14-443)

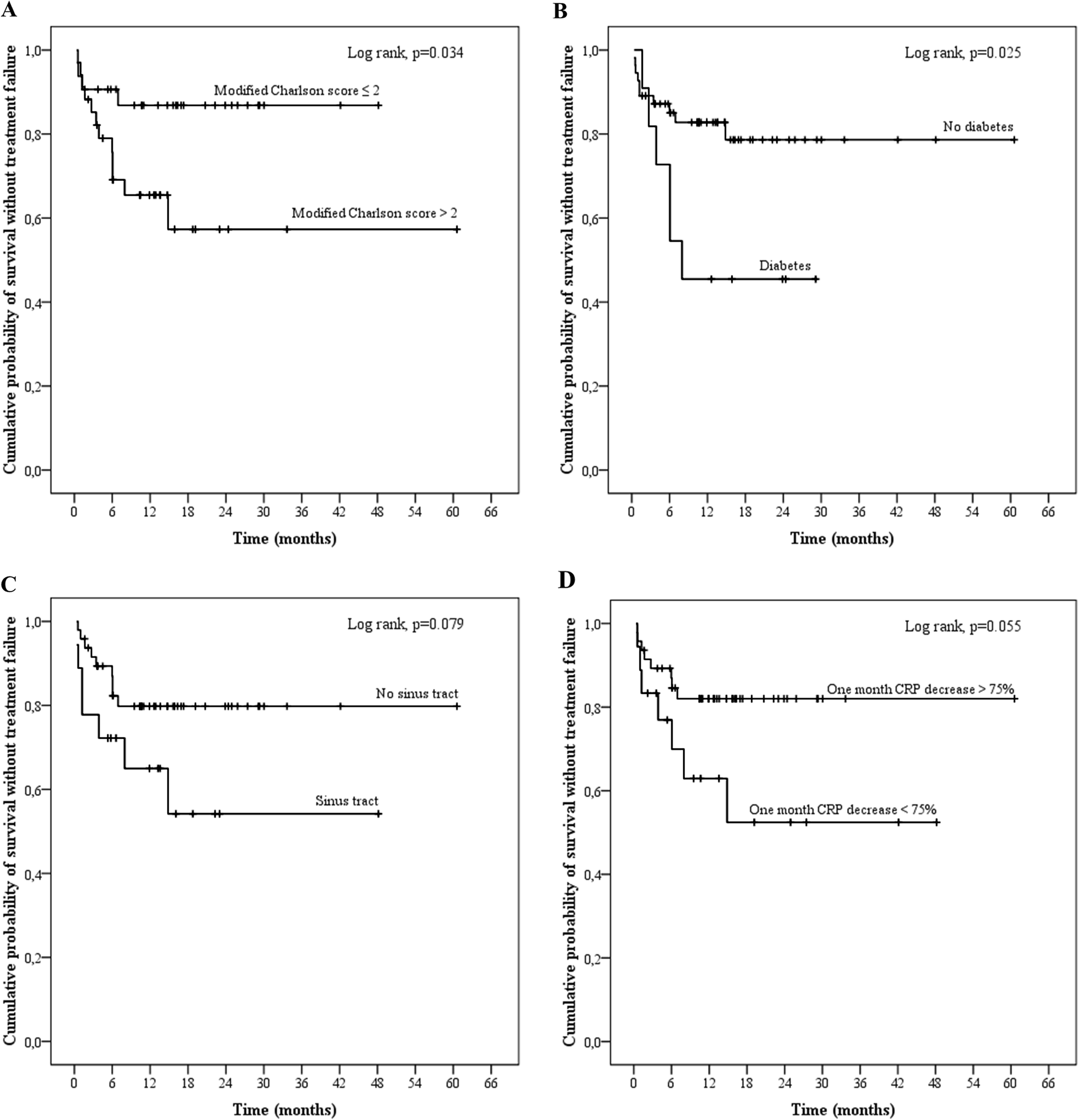

Supplement: Supplementary file 1 — Authors’ original file for figure 1 [file 12879_2014_3746_MOESM1_ESM.tif]
